# Study of conformational changes and protein aggregation of bovine serum albumin in presence of Sb(III) and Sb(V)

**DOI:** 10.1371/journal.pone.0170869

**Published:** 2017-02-02

**Authors:** Marcelo Verdugo, Jorge Ruiz Encinar, José Manuel Costa-Fernández, Mario Menendez-Miranda, Diego Bouzas-Ramos, Manuel Bravo, Waldo Quiroz

**Affiliations:** 1 Instituto de Química, Pontificia Universidad Católica de Valparaíso, Valparaíso, Chile; 2 Department of Physical and Analytical Chemistry, Faculty of Chemistry, University of Oviedo, Oviedo, Spain; Islamic Azad University Mashhad Branch, ISLAMIC REPUBLIC OF IRAN

## Abstract

Antimony is a metalloid that affects biological functions in humans due to a mechanism still not understood. There is no doubt that the toxicity and physicochemical properties of Sb are strongly related with its chemical state. In this paper, the interaction between Sb(III) and Sb(V) with bovine serum albumin (BSA) was investigated in vitro by fluorescence spectroscopy, and circular dichroism (CD) under simulated physiological conditions. Moreover, the coupling of the separation technique, asymmetric flow field-flow fractionation, with elemental mass spectrometry to understand the interaction of Sb(V) and Sb(III) with the BSA was also used. Our results showed a different behaviour of Sb(III) vs. Sb(V) regarding their effects on the interaction with the BSA. The effects in terms of protein aggregates and conformational changes were higher in the presence of Sb(III) compared to Sb(V) which may explain the differences in toxicity between both Sb species in vivo. Obtained results demonstrated the protective effect of GSH that modifies the degree of interaction between the Sb species with BSA. Interestingly, in our experiments it was possible to detect an interaction between BSA and Sb species, which may be related with the presence of labile complex between the Sb and a protein for the first time.

## Introduction

Antimony is widely used in the manufacture of different goods, such as flame retardants, catalysers, munitions and as an anti-parasitic drug against Leishmaniasis[1]. Additionally, one of the main sources of Sb pollution comes from the wear on vehicle braking systems which generates atmospheric particulate matter (APM) with high Sb content[2,3]. High levels of Sb enrichment in APM have been reported in Tokyo[2], Buenos Aires[4] and Valparaiso[5], finding a significant Sb enrichment in the blood of workers exposed to heavy vehicle traffic[6]. Recent studies have reported the toxic behaviour of antimony. As an example, in a recent study it has been shown that Sb(V) and Sb(III) are able to penetrate the erythrocyte membrane to reach the cytoplasm[7]. Chronic exposure to Sb can cause irritation of the eyes and skin and even cancer of the kidney, liver and lungs[8].

Sb toxicity mechanisms have been superficially studied so far. Most findings regarding Sb toxicity are based on thermodynamic similarities between Sb and As, as well as a series of inconclusive studies in living organisms [9]. For example, it has been proposed that Sb leads to protein aggregation, chaperone inhibition, and DNA inhibition [10,11]. There are also very few studies on the Sb reactivity in cell systems. Our own research group has found that Sb(V) reduces to Sb(III) in human erythrocytes, generating redox imbalance, decreasing the GSH/GSSG ratio and altering the activity of GPx and SOD, parameters associated with oxidative stress[12]. Evidence has also been found for the reduction of organic compounds containing Sb(V) used to treat Leishmaniasis and to produce ROS in the human macrophage cell line[13].

Obviously, the potential toxicity of Sb is related to the reactivity of its species and their redox effects. However, there are many other effects on a more macroscopic level associated with cell damage that must be considered in order to attain a more objective judgement of the toxicity of Sb and its species. One effect on a macromolecular level that is generated by the presence of metals and metalloids is protein aggregation [14,15]. In this process, hydrophobic interactions and hydrogen bridges cause the loss of the initial stability of the protein, thus changing its native structure. Redox alterations may also occur with disulphide bonds and sulphide-exchange, generating structural changes that can lead to the loss of protein functionality [16].

These aggregation mechanisms and conformational changes in proteins induced by different metal species have been studied using several different techniques, such as intrinsic or extrinsic molecular fluorescence with dyes, light scattering, gel electrophoresis, X-ray diffraction, circular dichroism, NRM and microscopy [17–20]. One of the most interesting model for the study of protein aggregation is albumin, mainly because of its properties and important biological functions, which are determined by its 17 pairs of sulfhydryl groups forming disulphide bridges and one free sulfhydryl group in position Cys-34, giving it high structural and reactive stability and making it the main antioxidant barrier in the plasma medium [21]. It has been used as a model for aggregation studies by means of thermal induction[22,23] and the presence of metals[24,25], showing evidence of the formation of dimerization, amorphous aggregates, proto fibres and metal coordination. In this context, there are many scientific studies that conclude that arsenic generates aggregation [26] and conformational changes [27,28] in protein. Even though it exist clear thermodynamic similarities between As(III) and Sb(III), the toxicity and carcinogenic properties of Sb are still in debate[29].

In order to shed some light over such general questions, it would be interesting to evaluate first the individual effects. Unfortunately, a systemic study of the effects of Sb(V) and Sb(III) on protein aggregation is still lacking. The objective of the present study was therefore to investigate the interactions between the inorganic species Sb(III) and Sb(V) with the protein BSA in vitro, under physiological pH. Several complementary spectroscopic tools have been used to detect eventual conformational changes of the BSA molecules, or protein aggregations, after interaction with the inorganic Sb species, which may be responsible for the toxicity of Sb in vivo.

## Experimental

### Reagents

All chemicals were of analytical grade and used as received. Deionized ultrapure water (resistivity 18.2 MΩcm) was used throughout the work. Antimony(III) oxide (99% purity), Potassium hexahydroxoantimonate(V) (99% purity) and Antimony standard for ICP 1000 mg L^-1^ were purchased from Sigma Aldrich (Buchs, Switzerland). The Sb(V) stock solutions (100 mg L^-1^) were prepared by dissolving an appropriate amount of the respective compounds in deionized water. These solutions were then stored in the dark at 4°C until use. Lower concentrations of Sb standards were prepared daily by diluting the stock solutions with deionized water. Bovine serum albumin (BSA) lyophilized powder (96% purity) and reduced L-Glutathione (GSH) (98% purity) were purchased from Sigma-Aldrich (St. Louis, MO, USA) and stored at 4°C. BSA was dissolved in 0.05 M phosphate buffer saline solution (PBS) and preserved at 4°C for later use. Ammonium acetate was used to prepare a 9 g L^-1^ buffer solution (pH = 7.4) to be used as the carrier solution in the asymmetric flow field-flow fractionation (AF4) instrument. All reagents occupied were of analytical grade. Sb(III) standards were prepared daily before each analysis.

### Spectroscopic characterisation

Fluorescence steady-state analysis and synchronous fluorescence spectra were performed using a Perkin Elmer LS-50B luminescence spectrophotometer (Beaconsfield, England) with a 3mm quartz cell at room temperature with excitation and emission slit of 20 nm. For synchronous fluorescence individual aliquots, were analysed and later discarded. The emission spectra were registered varying both the excitation and emission wavelengths between 240 to 320 nm, in order to obtain only the spectra corresponding to the tryptophan (Trp) residue of BSA, ensuring a constant wavelength difference (Δλ) of 60 nm between the excitation and emission wavelengths, and at a speed rate of 200 nm min^-1^. The maximum fluorescence intensity emission of Trp was obtained at the Exc/Em pair: 277/337 nm.

Circular dichroism (CD) spectra were measured using a J-815 spectropolarimeter (Jasco Corporate, Tokyo, Japan) with a 1.0 cm quartz cell at 37°C under constant nitrogen flush. The circular dichroism analysis was performed with a 1.0 cm quartz cell and equipped with a Jasco Peltier temperature controller set at 37°C (Model JWJTC). The scan speed was set at 100 nm min^-1^ and each spectrum was collected 3 times after measurement of a blank sample of 0.05 M PBS treated under the same conditions as the samples. Prior to all analysis the instrument was purged with nitrogen gas for 10 minutes. All the samples analysed by circular dichroism were incubated at 37°C for 24 and 72 hours. Mathematical analysis was performed using the SELCON3 algorithm, allowing comparison of the motif percentages on the secondary structure of the BSA.

The elemental analysis of S and Sb was carried out with an ICP-QQQ system (Agilent 8800, Tokyo, Japan). The simultaneous elemental-specific nature of the triple quad (ICP-QQQ) detector allowed the interference-free detection, of co-occurring elements present in the samples under study. Oxygen was introduced in the reaction cell at a flow rate of 0.35 mL min^-1^ and so S and Se were detected in mass shift mode (^32^S^+^→^48^SO^+^) [30]. Conversely, Sb was measured in on-mass mode (^121^Sb^+^→^121^Sb^+^). The integration time for each of the targeted isotopes ^32^S and ^121^Sb was 100 ms. The ICP-QQQ operation conditions were daily optimised using a tuning solution described in **Table A in S1 File**. Total S and Sb determination was performed by external calibration, injecting elemental standards online by flow injection analysis (FIA) with a six port injection valve (Rheodyne, IDEX Health & Science, Germany). Data acquisition and storage were carried out using the specific software of each instrument.

### Speciation analysis

Antimony speciation was achieved through anionic-exchange-HPLC-ICP-MS with a PRP-X100 column (Hamilton, USA) and an Agilent 1100 HPLC system (Agilent Technologies, Darmstadt, Germany). The isocratic program used consisted of Ethylenediaminetetraacetic acid (EDTA) 20mM as mobile phase at a flow rate of 1.5 mL min^-1^.

### Separation of protein aggregates

The separation of protein aggregates was conducted with an AF4 system AF2000 Focus model from Postnova Analytics Inc. (Landsberg, Germany) coupled on line with a fluorescence detector (Agilent 1260 infinity fluorescence detector, Darmstadt, Germany), operated at an excitation wavelength of 280 nm and an emission wavelength of 350 nm, and with the previously described ICP-QQQ detector. During the AF4 separation, regenerated cellulose membranes with 10 kDa molar mass cut-off were used as accumulation walls. The trapezoidal AF4 channel was 27.5 cm in length and a 500 μm thick spacer was employed. The experimental conditions of the AF4 system were optimised to be able to separate the different structures of BSA: monomer, dimer, trimer and oligomer which are showed in **Table B in S1 File**. The carrier solution used was filtered with cellulose nitrate membrane disc filters (Merck Millipore Co.) with a 0.1 μm pore before analysis. Integration of the fractographic peaks was performed using the MassHunter software (Agilent).

Alternatively, separation of protein aggregates by size exclusion chromatography (SEC) coupled on line to ICP-MS was also performed. For such purpose, an Agilent 1100 (Agilent Technologies, Darmstadt, Germany) was here employed. For SEC experiments, a Superdex 200 10/300 column was selected and the mobile phase composition was: ammonium acetate 50 mM at 0.6 mL min^-1^.

### Preparation of Antimony(III) oxide solution

Due to the low stability and low solubility of Sb(III), most studies involving such species have been carried out with its tartrate standard. However, the use of a tartrate standard in aqueous solution is not appropriate for understand Sb(III) toxicity studies as it is an anion that does not represent the natural chemical form in which Sb(III) is found in the atmosphere. Typically, Sb(III) in the environment appears as its oxide, Sb_2_O_3_, a compound of low water solubility and whose equilibrium in aqueous solution generates the species Sb(OH)_3_[31]. Since the objective of this study is to evaluate the effects of inorganic Sb(III) as it occurs in aqueous solution, it was decided to prepare solutions of Sb(OH)_3_ from its oxide, Sb_2_O_3_. Due to the low solubility of Sb_2_O_3_[32], the standard was prepared from an aqueous solution saturated in Sb_2_O_3_. In order to avoid re-oxidation of the Sb(III) in the presence of oxygen, purified deoxygenated water was used.

The determination of Sb concentration in the saturated aqueous solution, was performed by external calibration with Sb quantification by ICP-MS. The redox stability of Sb(III), was monitored using the HPLC-ICP-MS hybrid technique. All saturated solutions were filtered at 20 μm before analysis.

### Incubation of BSA with Sb species

BSA standards were prepared in PBS at pH 7.4 and incubated at 37°C with Sb at different concentrations and, in some experiments, with GSH. Samples containing Sb were prepared ensuring protein-metal molar ratios [BSA]:[Sb] 1:10 and [BSA]:[Sb] 1:2.5. The samples with GSH were prepared at molar ratios [BSA]:[Sb]:[GSH] 1:10:30 and [BSA]:[Sb]:[GSH] 1:2.5:7.5. Each sample was prepared by duplicate. The samples prepared for thermal protein aggregation studies were incubated for 3 hours at 58°C in a thermostat bath.

BSA incubation experiments were performed at a final concentration of 0.75 μM in 0.05 M PBS for fluorescence spectrometry and circular dichroism, respectively. Both BSA concentrations were selected according to its best signal/noise ratio. It is worth noting that the BSA concentration in the AF4-ICP-QQQ analysis of all the BSA-Sb incubation assays was 6.0 μM, which is higher than that used in the AF4 optimisation assays (0.75 μM), in order to allow detection of the different BSA structures: monomers, dimers, trimers and oligomers.

## Results and Discussions

### Conformational changes in BSA: Effects of Sb(III) and Sb(V) over conformational changes of BSA

A study has been made in order to investigate if, similarly to previous reports about arsenic toxicity, the presence of antimony (V) and antimony (III) could produce conformational changes on the BSA molecules, which may be responsible for the toxicity of antimony in vivo. Thus, in order to evaluate the eventual changes that may arise by the presence of Sb(III) and Sb(V) species in proteins, in vitro assays were conducted using bovine serum albumin (BSA) protein model.

Synchronous fluorescence is a simple and effective method to study the micro-environment of amino acid residues by measuring the emission wavelength shift. Therefore, it was used here to observe the eventual fluorescent changes in tryptophan (Trp) residue, and thus to eliminate the Raleigh signal. The registered BSA excitation-emission matrix in PBS at pH 7.4 is shown in **Figure A in S2 File**. If a protein aggregation occurs, significant changes in the fluorescence signal form Trp should be observed, such as a dampening and/or a shift in the Trp spectrum towards shorter wavelengths (blue-shift). These fluorescence changes if they occurs, may demonstrate changes in the polarity of the medium surrounding the amino acid residue, as well as changes in quantum efficiency[17].

During incubation of the BSA with different amounts of Sb(III) and Sb(V), aliquots were taken and their fluorescent spectra were registered every 15 minutes for 2 hours. These aliquots were finally discarded. In this way it was ensured to minimize the eventual effects produced by a continuous UV radiation over the fluorescent intensity of the Trp. It is worth noting that the continuous monitoring of the Trp fluorescence led to a significant signal decrease, approximately 10% in one hour of exposure, demonstrating that Trp is highly photosensible.

In order to observe the differential effect on the BSA generated by the Sb species, the synchronous fluorescence spectra were normalized with the maximum fluorescence of the Trp spectrum at the initial intensity of the control sample (BSA standard).

**Fig 1:**
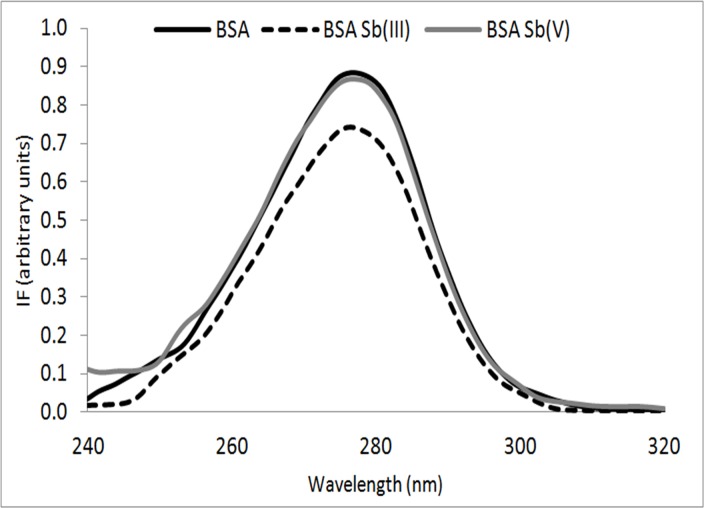
Synchronous fluorescence spectra for BSA (line in black), BSA-Sb(III) (dashed line), BSA-Sb(V) (line in gray) incubated at 37°C for 2 hours. Molar ratio [BSA]:[Sb] 1:10, [BSA] = 0.75 μM.

As shown in **Fig 1** for the BSA samples incubated with Sb(III), a significant decrease in the fluorescence of the Trp residue, as compared with the spectra obtained for a pure BSA solution (control solution), was observed. These phenomena may imply that the interaction of Sb(III) with BSA may decrease the polarity near the Trp residues (resulting in a more hydrophobic environment for the Trp residues), thus affecting the quantum efficiency. In contrast, this effect was not observed when the BSA incubation was performed with the same concentration of Sb(V). It is important to mention that by means of dilution tests it was possible to determine that there is no internal filter in the fluorescence assays performed at the concentrations of BSA (**Figure B in S2 File**), BSA-Sb(III) (**Figure C in S2 File**) and BSA-Sb(V) (**Figure D in S2 File**) (BSA: Sb 1:10 [BSA] = 0.75 μM).

**Fig 2:**
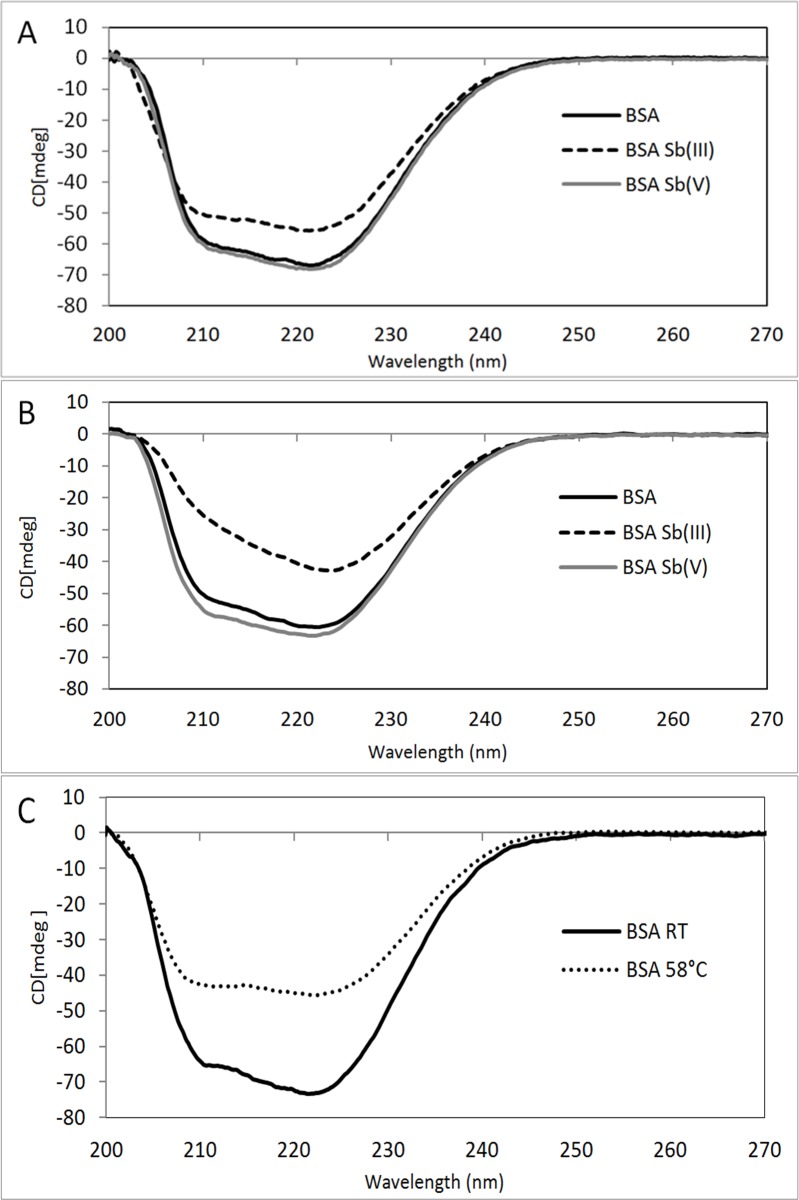
Circular Dichroism spectra for BSA (line in black), BSA-Sb(III) (dashed line) and BSA-Sb(V) (line in gray). (A) 24 hours and (B) 72 hours of incubation at 37°C and (C) BSA at room temperature (black) and with heat-induced aggregation at 58°C for 3 hours (dotted line).

It is well known that far-ultraviolet circular dichroism (CD) spectrum reflects the spatial orientation of the peptide bond or the secondary structure of the protein. In order to support further the effect of Sb on conformational changes of BSA, far-ultraviolet CD of BSA was performed. The samples were prepared following the same procedure already described for the molecular fluorescence analysis, ensuring a protein-metal molar ratio of [BSA]:[Sb] 1:10. As shown in **Fig 2**, there are two negative bands in the far-UV region at 208 nm and 222 nm and a positive band at 215 nm, which are characteristic of the α-helical structure of a protein. The two negative peaks both contributed to an n→π^*^ transition for the peptide bond with α-helicity [17]. The CD spectra were taken after incubating the BSA samples with Sb for 24 and 72 hours, allowing observation of eventual changes in the secondary structure of BSA, mainly in the peaks associated with α-helix motifs found in the far UV at 208 nm and 222 nm [17]. In the near UV zone, no significant changes were seen in the registered CD spectrum, indicating that Sb does not generate significant changes in the tertiary structure of BSA.

Samples containing BSA showed no significant changes after 24 and 72 hours of incubation (**Fig 2A and 2B**). Only a small decrease was seen in the α-helix motif after 72 hours. Similar behaviour was also seen in the BSA samples with Sb(V), which is in agreement with the molecular fluorescence results, where Sb(V) did not cause significant changes in the protein.

Conversely, for the BSA samples incubated with Sb(III), significant changes can be seen, mainly in the α-helix and regular β-sheet motifs and random structures. After 24 hours of incubation, the regular α-helix motif decreases and is distorted, while the regular β-sheet motif increases. As can be seen in the results in **Table 1**, after 72 hours of incubation these structural changes increased, changing from the regular and distorted structure of the α-helix and random structures towards the regular β-sheet. The resulting conformational transition to β-sheet induced by Sb(III), can be due to a direct Sb(III)-BSA interaction or self-assembly of BSA, that lead to a more stable non-native structure.

**Table 1:** Percentage of secondary structure of BSA, BSA-Sb(III) and BSA-Sb(V) at 24 and 72 hours of incubation at 37°C, BSA at room temperature (RT) and with heat-induced aggregation at 58°C for 3 hours. [BSA] 0.75 μM. Molar ratio of [BSA]:[Sb] 1:10.

	BSA	BSA-Sb(III)	BSA-Sb(V)	BSA	BSA-Sb(III)	BSA-Sb(V)	BSA	BSA
	24 h	24 h	24 h	72h	72h	72h	RT	58°C
**Regular α-helix**	10.3 ± 0.1	7.3 ± 0.1	9.7 ± 0.1	9.0 ± 0.1	5.2 ± 0.1	9.6 ± 0.1	39.0 ± 0.1	21.7 ± 0.1
**Distorted α-helix**	10.2 ± 0.1	8.8 ± 0.1	10.2 ± 0.1	9.7 ± 0.1	7.3 ± 0.1	9.7 ± 0.1		
**Regular β-sheet**	10.8 ± 0.3	11.2 ± 0.1	13.6 ± 0.4	11.5 ± 0.3	16.6 ± 0.2	11.0 ± 0.1	15.9 ± 0.2	23.9 ± 0.3
**Distorted β-sheet**	10.8 ± 0.3	10.7 ± 0.1	10.1 ± 0.3	10.5 ± 0.3	10.5 ± 0.1	10.8 ± 0.1		
**Turns**	24.2 ± 0.1	25.4 ± 0.1	24.1 ± 0.1	24.4 ± 0.1	23.4 ± 0.1	24.8 ± 0.1		
**Random structure**	42.2 ± 0.1	45.5 ± 0.1	37.8 ± 0.1	42.7 ± 0.1	37.3 ± 0.1	44.5 ± 0.1	45.1 ± 0.1	54.4 ± 0.1

It is known that in a solution at 58°C conformational change of proteins occurs without the presence of metal ions. This condition was used as a positive control previously by Navarra et al.[25]. As can be seen in **Fig 2C** after a heat shock-induced aggregation by means of incubation at 58°C for 3 hours changes in the secondary structure, going from α-helix to β-sheet and random structures occurs. This results are consistent with previous research which reports structural changes when BSA is subjected to high temperatures, seen using circular dichroism, with the BSA changing from α-helix to β-sheet[22]. It is interesting to note that the profiles observed for the control for positive aggregation and the incubation with Sb(III) were quite similar, clearly suggesting that Sb(III) is also promoting BSA aggregation. On the other hand it must be considered that BSA is a globular type protein so we believe non structured aggregation instead of fibrillation may be the most plausible mechanism to explain results of **Fig 2.** However it has been reported that BSA [33] and lysozyme [34] can generate fibrillar structures in a few hours after thermal shock, this aggregation mechanism were evidenced by using electron microscopy (TEM), and atomic force microscopy (AFM) techniques to access and characterize the whole aggregation pathway.

### Conformational changes in BSA: Protective effect of GSH

Reduced glutathione (GSH) is a linear tripeptide of L-glutamine, L-cysteine, and glycine present in most animal cell systems in the range of 3–10 mM. GSH is an extremely important cell protectant. It directly quenches reactive hydroxyl free radicals, other oxygen-centered free radicals, and radical centers on DNA and other biomolecules [35,36]. In blood plasma GSH is found at around 10 mM with albumin close to 480 mM[37]. It is known that both species generate detoxification processes of cytotoxic agents due to their antioxidant characteristics [38]. For example, GSH is a ligand of metals such as Pb, Cu or Fe[39], protecting BSA from oxidative damage by metals and it can also generate adducts with BSA[40]. GSH also forms complexes with Sb(III) Sb(GSH)_3_, likely suggesting that it has a protective role against processes of conformational changes and BSA aggregation in the presence of Sb(III) and Sb(V)[41,42].

**Fig 3:**
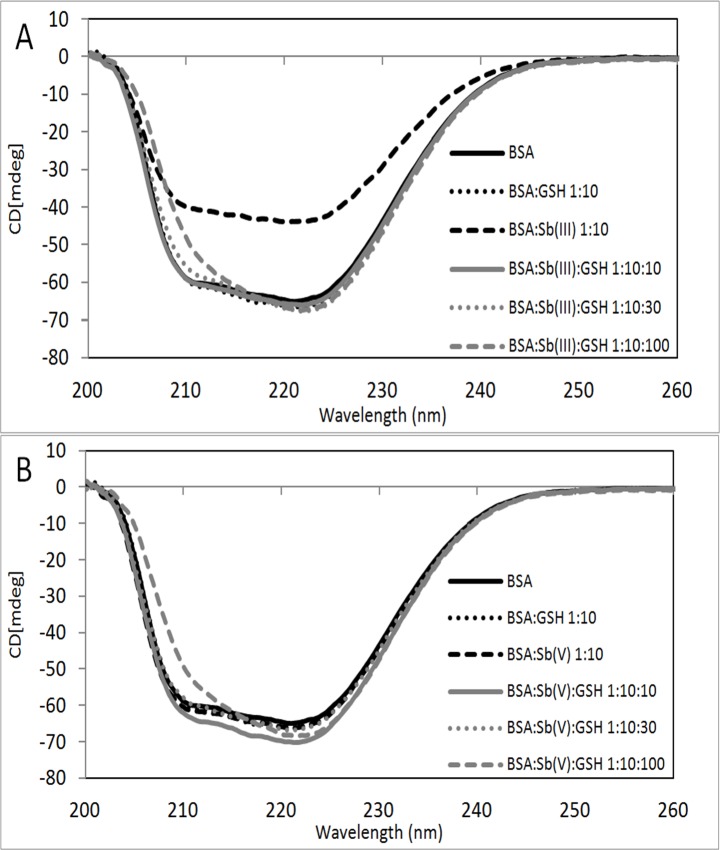
Circular dichroism spectra for BSA (line in black), BSA:GSH 1:10 (dotted line in black), BSA:Sb 1:10 (dashed line in black), BSA:Sb:GSH 1:10:10 (line in gray), BSA:Sb:GSH 1:10:30 (dotted line in gray) and BSA:Sb:GSH 1:10:100 (dashed line in gray). Incubated with Sb(III) (A) and Sb(V) (B) for 72 hours at 37°C. [BSA] 0.75μM.

In order to obtain experimental evidence of this hypothetical protective effect of GSH, BSA-Sb(III/V) incubations were performed in the presence of GSH and incubated for 72 hours at molar ratios of 1:10 for [BSA]:[Sb], adding variable molar ratios of GSH from 1:10:0 to 1:10:100 BSA:Sb:GSH. Then, samples were analysed by circular dichroism as previously reported. Results obtained are shown in **Fig 3**.

As shown in **Fig 3** the effect of Sb(III) on conformational changes of BSA are clearly diminished in the presence of GSH, already at the low molar ratios BSA:Sb(III):GSH of 1:10:10. The effect of Sb(V) on BSA structure is again no significant. It is interesting to note that when GSH ratio increases the secondary structure changes to α-helix. Results are shown in **Table 2**.

**Table 2:** Percentage of secondary structure of BSA, BSA-Sb(III) and BSA-Sb(V) with GSH at different molar ratios, incubated for 72 hours at 37°C and analysed with SELCON3. Molar ratio of [BSA]:[Sb] = 1:10. [BSA] 0.75μM.

	Regular α-helix	Distorted α-helix	Regular β-sheet	Distorted β-sheet	Turns	Random structures
BSA	9.9 ± 0.1	9.8 ± 0.1	11.6 ± 0.3	10.7 ± 0.3	24.3 ± 0.1	42.3 ± 0.1
BSA GSH 1:10	10.0 ± 0.3	10.4 ± 0.3	11.7 ± 0.5	10.5 ± 0.5	24.5 ± 0.1	40.8 ± 0.2
BSA Sb(III) 1:10	4.5 ± 0.1	7.1 ± 0.1	15.7 ± 0.1	10.4 ± 0.1	23.7 ± 0.1	39.7 ± 0.1
BSA Sb(III) GSH 1:10:10	9.3 ± 0.1	9.9 ± 0.1	13.2 ± 0.2	10.5 ± 0.2	24.7 ± 0.1	40.7 ± 0.1
BSA Sb(III) GSH 1:10:30	10.8 ± 0.3	10.7 ± 0.3	12.4 ± 0.4	9.1 ± 0.3	21.7 ± 0.1	33.2 ± 0.2
BSA Sb(III) GSH 1:10:100	11.0 ± 0.1	10.8 ± 0.1	9.7 ± 0.1	9.9 ± 0.1	25.4 ± 0.1	42.1 ± 0.1
BSA Sb(V) 1:10	9.3 ± 0.1	9.9 ± 0.1	14.4 ± 0.4	10.3 ± 0.3	23.7 ± 0.1	37.8 ± 0.1
BSA Sb(V) GSH 1:10:10	10.3 ± 0.1	10.6 ± 0.1	12.9 ± 0.2	9.4 ± 0.2	22.1 ± 0.1	34.5 ± 0.1
BSA Sb(V) GSH 1:10:30	9.7 ± 0.3	10.2 ± 0.3	12.1 ± 0.4	10.4 ± 0.4	24.5 ± 0.1	40.4 ± 0.2
BSA Sb(V) GSH 1:10:100	11.5 ± 0.1	10.8 ± 0.1	9.8 ± 0.1	10.1 ± 0.1	24.3 ± 0.1	39.5 ± 0.1

It is clear from results shown in **Table 2** that the increase in the molar ratio of GSH leads to changes in the secondary structure of the BSA, mainly in the regular β-sheet motif. These changes can also be explained by the generation of adducts. For the samples incubated with Sb(III) or Sb(V), GSH has a protective effect against conformational changes generated by Sb species, but not fully return to the BSA secondary structure attained by the BSA with GSH.

**Fig 4:**
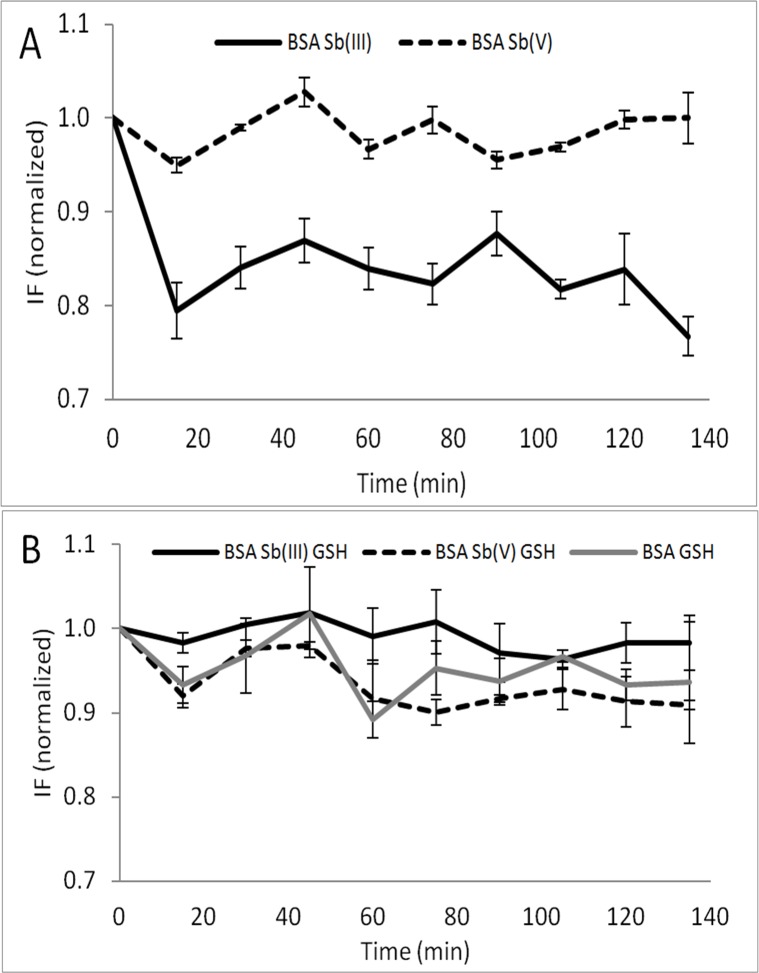
Fluorescence intensity of BSA in the presence of Sb(III) (line in black) and Sb(V) (dashed line). (A) in the absence of GSH. (B) in the presence of GSH. BSA GSH (line in gray). [BSA]:[Sb]:[GSH] 1:10:30. [BSA] = 0.75μM. Exc/Em 277/337 nm. (n = 2, uncertainty corresponds to 1 SD).

Next, the fluorescence spectra of BSA in the presence of Sb (with and without the presence of GSH) at different incubation times were measured to detect any BSA fluorescence quenching caused by the interaction with Sb. In order to compare the BSA fluorescence signals in the presence/absence of Sb and to confirm the protective effect of GSH on the conformational changes described previously by Sb(III) (see **Fig 3**), the intensity signals obtained of Trp were normalized with the fluorescence intensity of the sample at time zero and with the fluorescence intensity of BSA standard as a function of time. The effect of Sb on the fluorescence of BSA (**Fig 4**) and the protective effect generated by GSH against conformational changes in BSA were both investigated.

As can be seen in **Fig 4A**, the presence of Sb(III) produces a significant quenching on the BSA fluorescence (a 20% decrease on the fluorescence intensity was measured) which are similar results compared with those obtained in **Fig 1**, thus demonstrating an interaction between the Sb(III) with the protein affecting the environment of the Trp. However, no significant fluorescence quenching was observed when BSA was incubated with Sb(V). It is very interesting to note that the presence of GSH avoided the fluorescence quenching (**Fig 4B**), indicating again the protective effect of GSH. In fact, the normalized fluorescence maintained statistically similar to the fluorescence of BSA GSH over time in the presence and in the absence of both Sb(III) and Sb(V).

If we infer the hypothetical effect of Sb(V) and Sb(III) on protein aggregation in in-vivo conditions it can be state that based on fluorescence and circular dichroism (**Figs 3 and 4**) results that interaction between Sb(III)-BSA does not occur because of the presence of GSH which inhibit the BSA aggregation of Sb(III) (**Table 3**). On the other hand Sb(V) generate no-effect at all.

**Table 3:** BSA aggregation percentage in the presence of Sb(III), Sb(V) and GSH incubated for 72 hours at 37°C. Molar ratio of [BSA]:[Sb]:[GSH] 1:2.5:7.5. [BSA] = 6.0 μM. Results are given as relative percentages out of the total area obtained from the ^32^SO signal obtained by AF4-ICP-QQQ.

	% Monomer	% Dimer	% Trimer	% Oligomer	% ∑ Aggregates
BSA	83.2 ± 0.8	7.6 ± 0.2	1.4 ± 0.1	7.9 ± 0.5	16.8 ± 0.8
BSA GSH	87.7 ± 0.1	6.0 ± 0.2	0.9 ± 0.1	5.4 ± 0.1	12.3 ± 0.1
BSA Sb(III)	80.7 ± 0.4	8.0 ± 0.1	1.8 ± 0.1	9.6 ± 0.3	19.3 ± 0.4
BSA GSH Sb(III)	84.6 ± 0.1	5.6 ± 0.1	0.8 ± 0.1	8.9 ± 0.1	15.4 ± 0.3
BSA Sb(V)	80.9 ± 0.7	8.5 ± 0.1	1.6 ± 0.1	8.9 ± 0.5	19.1 ± 0.7
BSA GSH Sb(V)	84.6 ± 0.2	5.9 ± 0.1	0.7 ± 0.1	8.8 ± 0.2	15.4 ± 0.2

However, in a recent article published by our research group we demonstrate that Sb(III) generate an effect over protein mobility in human kidney cells similar to those generated by As(III). About the efficiency of protein aggregation our results in **Table 3** shows that for both species is relatively similar where % of aggregation are 19.3±0.4 for Sb(III) vs. 19.1±0.7 for Sb(V). However if we consider conformational changes it is clear that the effect of Sb(III) is higher than Sb(V) as can be seen in **Fig 3** in CD spectra and **Fig 4A** fluorescence spectra. Again these results are consistent with our previously published results in in vivo conditions in kidney cells where we show that the effect of Sb(III) on the mobility of proteins is greater than the effect of Sb(V) which is related to protein conformational changes[43].

### Separation and detection of BSA aggregates by SEC and AF4

For the study of protein aggregation, it is necessary to use techniques with mild separation conditions and the minimum sample distortion. For this reason we decided to use SEC and AF4, coupled on line to an ICP-QQQ detector, as it provides sensitive detection of both the metal under study (Sb) and the protein through the detection of the S present in its Cys and Met residues[44].

**Fig 5:**
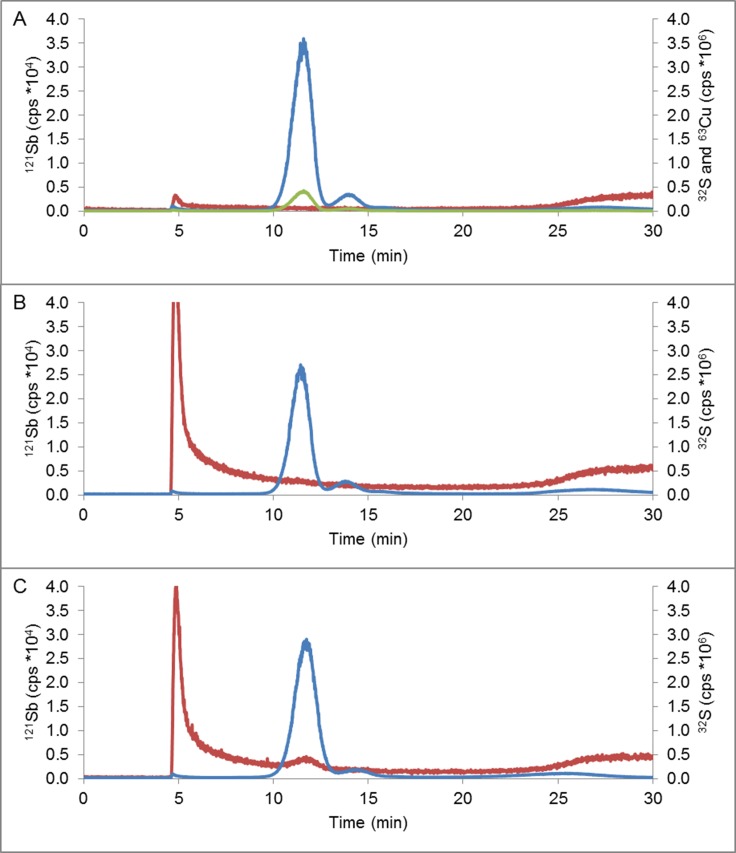
ICP-QQQ fractograms. (A) BSA at room temperature. (B) BSA with Sb(III) at room temperature without incubation. (C) BSA with Sb(III) incubated by 72 hours at 37°C: ^32^S (line in blue), ^121^Sb (line in red) and ^63^Cu (line in green). Molar ratio [BSA]:[Sb] 1:2.5. [BSA] = 6.0μM.

A first approach was performed by SEC, trying to separate the three different coexisting structures of a BSA standard (monomer, dimer and oligomer). Unfortunately, after several attempts we were able only to detect the monomeric structure of the protein eluting at 22 minutes (**Figure E in S2 File**). In contrast, when the same sample was analysed by AF4, the monomer, dimer, trimer and even oligomer isomers of the BSA standard (**Fig 5A**) could be observed. These results clearly prove the better performance of AF4, as compared to SEC, for the analysis of these BSA macrostructures.

In order to minimise the likely induction of BSA aggregation during the AF4 process, the separation conditions were optimised. These include injection time and cross flow conditions. The optimization described in **Table C in S1 File** shows the relative aggregation percentage from the total detected species eluted from the separation channel under different AF4 separation conditions (test 1 to test 5, see **Table C in S1 File**). It was found that the optimum conditions correspond to the injection of 200 μL of a sample, (4 min injection time), followed by 10 min of constant cross flow at rate 2.5 mL min^-1^ and then 10 min of cross flow decay. This AF4 program allows the separation and detection of monomer, dimer, trimer and oligomer species of BSA, while showing low aggregation percentage in the eluted species. All fractograms presented in this work were obtained under this optimum AF4 conditions.

Finally, we wanted to demonstrate that the AF4-ICPQQQ coupling was able to detect changes in BSA aggregation. For that purpose, we compared the profile obtained for BSA incubated at room temperature and at high temperature (58°C), which is known to induce thermal aggregation [25]. As can be seen in **Figure F in S2 File** after incubation at high temperature, the peak corresponding to the monomer decreased whilst the peak corresponding to the dimer increased accordingly. This is clear evidence that the AF4-ICPQQQ can be used to monitor changes in protein aggregations.

### Sb(III) and Sb(V) effects over BSA aggregation

Once the AF4 system was optimized and validated, the effect of Sb(III) and Sb(V) on the degree of BSA aggregation was studied by the hybrid technique AF4-ICP-QQQ. Results obtained are collected in **Table 3**.

These results show that both Sb(III) and Sb(V) increase the percentage of aggregation BSA. For example the percentage of aggregation of BSA detected in the fractograms (i.e. obtained from the relative area of the aggregate peak as compared to the total area of BSA species eluted from the AF4 channel and detected by the ICP-QQQ) increased from 16.8 ± 0.8% to 19.3 ± 0.4% (n = 3, 1 SD) in the presence of Sb(III) generating an increase in all fractions of BSA aggregates especially the fraction of the oligomer. Similarly, Sb(V) led to an increase in the BSA relative aggregation from 16.8 ± 0.8% to 19.1 ± 0.7%.

Moreover, the protective effect of GSH on BSA aggregation in the presence of Sb species was analysed in **Table 3**. The results show that the natural BSA aggregation decreases from 16.8 ± 0.8% to 12.3 ± 0.1% in the presence of GSH, indicating a protective effect against aggregation induced at 37°C. For the samples incubated with Sb, a protective effect was also seen against aggregation in the presence of GSH. For example, the BSA aggregation percentage in the presence of Sb(III) increases from 16.8 ± 0.8% to 19.3 ± 0.4%, and decreases to 15.4 ± 0.3% when adding GSH to the mixture, indicating the protective effect of GSH for the BSA aggregation induction by Sb(III). In the case of Sb(V), a similar trend can be seen, where the BSA aggregation of 16.8 ± 0.8% increases to 19.1 ± 0.7% in the presence of Sb(V) and then falls to 15.4 ± 0.2% when GSH is added to the mixture. These aggregates structures were impossible to detect by using CD or molecular fluorescence techniques, in which signals of aggregate structures were overlapped with the signal of the monomer which is predominant (about 85%) in the mixture.

GSH has been reported to stabilise the native structure of BSA through the formation of BSA-GSH adducts[45]. The mechanism proposed by some researchers relies on the formation of adducts between protein and GSH mainly through -SH/S-S sulfhydryl-group exchange reactions[46]. In the case of BSA, this adduct formation can occur due to the free cysteine residue present[47]. The equilibrium between the free Cys and the formation of disulphide bonds can be affected for example by the temperature of the medium, leading to thermal denaturation. Under such altered conditions, proteins can become aggregated as dimers and oligomers, leading to irreversible structural changes and aggregation[46]. These conformational changes not only occur when covalent bonds are affected, such as disulphide bonds, also can be occur due to environmental changes that carry to lacking of electrostatic interactions and hydrogen bonds that generate stability in the native protein structure in a intricate network of interaction between side chains and the environment[48]. Some species are considered as -SH reaction blockers, such as cysteine and reduced glutathione [46,49]. This may explain why the GSH protects against BSA aggregation as observed by the AF4-ICP-QQQ data provided in **Table 3**.

About the effects of Sb species on denaturation or folding to the best of our knowledge there is no reports about Sb species or metalloids such as As species which generate those particular effects on BSA protein. Most of the studies reported in literature are focused on metals or transition metals such as Ca^2+^, Cd^2+^, Zn^2+^ or Co^2+^ [50] which are cations in solution. However in the case of metalloids such as As o Sb, all of their species are anions or neutral species in solutions such as H_2_AsO_4_^-^ for As(V), Sb(OH)_6_^-^ for Sb(V) or Sb(OH)_3_ for Sb(III) so it is no correct to make extrapolations from those studies to this case. Finally it is important to mention that in this study it was not included the chirality factor because BSA protein used was just the natural racemic mixture. For example it was described that Cd^2+^ presents stronger interactions with L-peptides vs. D-Peptides[51]. However as far as knowledge goes there are no reports about the interactions of Sb(V) or Sb(III) with different chiral forms of BSA or smaller molecules such as peptides and the weight of the chiral factor over the interaction of Sb(V) or Sb(III) is still an unsolved problem.

### Separation and detection of Sb-S BSA complexes. Assessment using AF4-ICPQQQ

Previously has been reported complexes of Sb and biomolecules such as glutathione by ^1^H NMR and ESI-MS, being the complex stoichiometry 1:3[41,42]. The formation of other Sb complexes has been observed as well, such as Sb-adenine nucleosides (observed by circular dichroism, nuclear magnetic resonance ^13^C and ^1^H NMR[52]), Sb-guanine nucleosides complexes (observed by by ^1^H NMR), and antimony with BPR (bromopyrogallol)[53] (observed by ESI-MS and spectrophotometry). The interaction of Sb-S for the complex Sb(GS)_3_ has been reported to show a high stability constant, even more stronger than for Cd(II), but with a high kinetic lability at physiological pH (9000 s^-1^) [41].

In order to support further the hypothetical interaction previously observed between the Sb species and the BSA protein, SEC-ICP-MS was used for the analysis of the samples after incubation of BSA and Sb(III). The corresponding chromatogram is shown in **Figure E in S2 File**. As can be seen, BSA eluted at around 22 minutes. However, the elution of Sb occurs after 30 minutes separately, indicating that the formation of any potential complex between Sb(III) and BSA could not be detected. However, based on those results it is not possible to determine if either the Sb-BSA complexes are not formed or they are formed but destroyed during the chromatography process due to their high lability. In fact, we had previously observed that some unspecific interactions between the polymeric SEC stationary phase are likely to occur, leading to destruction of labile complexes [54]. Therefore, we decided to resort again to the AF4-ICP-QQQ. **Fig 5A** shows the analysis of BSA standard at room temperature monitoring the signals of S and Cu, which is intrinsically present in all structures of BSA standard. Moreover, it is shown that the background ^121^Sb signal does not change when BSA elutes, confirming that the elution of BSA does not interfere in the detection of ^121^Sb. Alternatively, **Fig 5B** shows the fractogram obtained for a BSA standard that was spiked with Sb(III) just before the injection (without any incubation). The excess of Sb eluted at a huge peak at the void. It is interesting to note that Sb(III) did not generate any significant effects in the BSA aggregation in this case as it seems that there is no time enough to produce any Sb-BSA interaction. Moreover it is again clear that the BSA elution does not produce any distortion in the tail of the Sb signal. Besides, it was evaluated the interaction BSA-Sb after incubation for 72 hours at 37°C (**Fig 5C**). In this case, it was possible to detect a small Sb signal that eluted together the BSA monomer in presence of Sb(III), which could be ascribed to the labile complex Sb-BSA. Similar results were obtained after incubation with Sb(V).

Unfortunately, the BSA-Sb interaction cannot be evaluated quantitatively because of the low amount of Sb detected in the BSA monomer which is very close to the detection limit of the employed methodology. It is known that Sb can form labile complexes with various organic molecules such as peptides with -SH groups like GSH [41]. However, from the results obtained cannot be concluded if the formation of the BSA-Sb complex is almost negligible or it is more significant but impossible to be detected properly due to its extreme lability. On the other hand, it is mandatory to know the stoichiometry, binding energy and interaction mechanism of the Sb(V)-BSA and Sb(III)-BSA complexes which cannot be obtained by AF4-ICP-MS. For this aim it is mandatory the use of techniques such as ESI-MS, Time Resolved Fluorescence, isothermal titration calorimetry, multiple spectroscopy, molecular modeling, zeta-potential, resonance light scattering, conductometry and molecular modeling[55–59].

The BSA-Sb complex was detected only in the monomer and not in the aggregated structures. The monomer fraction corresponds to approximately 85% of the BSA and, therefore, a complex with other BSA fractions may be present below the detection limit of the technique. It is interesting to note here that to the best of our knowledge this is the first time that the complex formed between Sb and a protein (BSA) could be preserved and unambiguously detected. There is no direct evidence of Sb binding sites on protein. However most of the researchers on this field believe that the most plausible protein binding site for Sb must be with–SH group based on the complexes which form with glutathione peptide[41].

As a synthesis based on all the results shown the most logical inference would be that in in vivo conditions the peptide GSH should inhibit the bond between Sb(III) or Sb(V) with BSA. However, in a recent article our research group demonstrate that Sb(III) generate an effect over protein mobility in human kidney cells similar to those generated by As(III)[43] so we have direct and in vivo empirical evidence that shows that at least Sb(III) interacts with proteins so that this potential inhibition of GSH is not enough at least for human kidney cells.

## Conclusion

Based on the evidence gathered in this study, it can be stated that Sb(III) generates conformational changes in the structure of the protein BSA, while Sb(V) does not. In addition, GSH is a protective agent against the effects of Sb on protein conformational changes.

It can also be stated that both Sb(III) and Sb(V) are able to promote slightly BSA aggregation. In addition, it was determined that GSH acts as a protective agent to the BSA aggregation, as was observed with conformational changes of BSA. Based on our results, we can affirm the existence of a complex BSA-Sb, which it cannot be evaluated quantitatively due to the low signal detected along with BSA monomer.

Finally our results showed that GSH generates a protective effect to conformational changes and aggregation of BSA which can be explained by modifications on the degree of interaction between Sb(III) or Sb(V) with BSA. In this point it is important to continue this work considering in vivo experiments.

It is a projection of this work to determine the type of interaction that exerts GSH over BSA-Sb complex as well as to determine its stoichiometry. The AF4-ICP-QQQ system proposed here seems to be an adequate tool to face such challenges. However we believe that future research must consider to evaluate the Sb(V)-BSA and Sb(III)-BSA complexes binding constant and stoichiometry through another techniques such as TEM, scanning electron microscopy (SEM), Time Resolved Fluorescence, Electrospray Mass Spectrometry or AFM could be very useful to obtain this information.

## Supporting Information

S1 File **Table A. ICP-MS parameters.** Optimal ICP-MS parameters for S and Sb measurement. **Table B. BSA separation.** Conditions of BSA separation by AF4. **Table C. AF4 conditions for the separation of BSA structures.** AF4 optimization conditions for the separation of BSA structures by AF4-ICP-QQQ ([BSA] = 0.75μM). Results are gven as individual relative percentage for each structure out of the total area obtained.
(ZIP)

S2 File **Figure A. BSA excitation emission matrix.** [BSA] = 0.75mM. BSA excitation emission matrix in PBS at pH 7.4. [BSA] = 0.75mM. **Figure B. Synchronous fluorescence spectra for BSA.** [BSA] = 0.75 μM. Synchronous fluorescence spectra for BSA at room temperature on dilution 1:1 (black line), 1:5 (red line), 1:10 (blue line), 1:20 (purple line). [BSA] = 0.75 μM. **Figure C. Synchronous fluorescence spectra for BSA in presence of Sb(III).** Synchronous fluorescence spectra for BSA in presence of Sb(III) at room temperature on dilution 1:1 (black line), 1:5 (red line), 1:10 (blue line), 1:20 (purple line). Molar ratio [BSA]:[Sb(III)] 1:10. [BSA] = 0.75 μM. **Figure D. Synchronous fluorescence spectra for BSA in presence of Sb(V).** Synchronous fluorescence spectra for BSA in presence of Sb(V) at room temperature on dilution 1:1 (black line), 1:5 (red line), 1:10 (blue line), 1:20 (purple line). Molar ratio [BSA]:[Sb(V)] 1:10. [BSA] = 0.75 μM. **Figure E. SEC-ICP-QQQ chromatograms of BSA incubated in presence of Sb(III).** SEC-ICP-QQQ chromatograms of BSA incubated by 72 hours at 37°C in presence of Sb(III) corresponding to ^32^S (line in black) and ^121^Sb (line in grey) signals. Molar ratio [BSA]:[Sb(III)] 1:10. [BSA] = 0.75 μM. **Figure F. ICP-QQQ fractograms of BSA.** ICP-QQQ fractograms of BSA at room temperature (black) and 58°C (grey) corresponding to the ^32^SO* signal. [BSA] = 0.75μM.
(ZIP)
