# Supplementary material for: Study of conformational changes and protein aggregation of bovine serum albumin in presence of Sb(III) and Sb(V)
Source: PLoS One. 2017 Feb 2;12(2):e0170869. doi: 10.1371/journal.pone.0170869 (PMC5289473; doi:10.1371/journal.pone.0170869)
Supplement: S1 File — Table A. ICP-MS parameters. Optimal ICP-MS parameters for S and Sb measurement. Table B. BSA separation. Conditions of BSA separation by AF4. Table C. AF4 conditions for the separation of BSA structures. AF4 optimization conditions for the separation of BSA structures by AF4-ICP-QQQ ([BSA] = 0.75μM). Results are gven as individual relative percentage for each structure out of the total area obtained. (ZIP) [file pone.0170869.s001.zip › TableA.docx]

**Table A. Optimal ICP-MS parameters for S and Sb measurement.**

|  |  |
| --- | --- |
| Radio frequency | 1550 W |
| Sampling Depth | 8.0 mm |
| Carrier gas flow rate | 0.85 L min^-1^ |
| Extraction lens |  |
| Extract 1 | -5 V |
| Extract 2 | -200 V |
| Omega | 10 V |
| Omega Bias | -100 V |
| Q1 bias | -5 V |
| Cell |  |
| Cell gas | O_2_ |
| Cell gas flow rate | 0.35 mL min^-1^ |
| Octapole Bias | -10 V |
| KED | -8 V |
