# Supplementary figures and images for: Study of conformational changes and protein aggregation of bovine serum albumin in presence of Sb(III) and Sb(V)
Source: PLoS One. 2017 Feb 2;12(2):e0170869. doi: 10.1371/journal.pone.0170869 (PMC5289473; doi:10.1371/journal.pone.0170869)

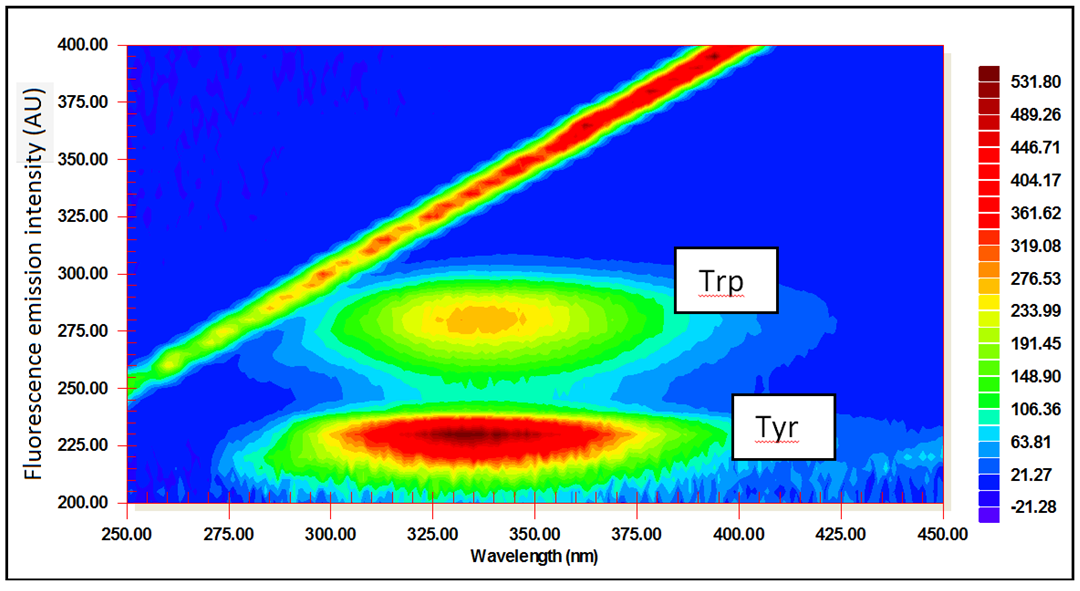

Supplement: S2 File — Figure A. BSA excitation emission matrix. [BSA] = 0.75mM. BSA excitation emission matrix in PBS at pH 7.4. [BSA] = 0.75mM. Figure B. Synchronous fluorescence spectra for BSA. [BSA] = 0.75 μM. Synchronous fluorescence spectra for BSA at room temperature on dilution 1:1 (black line), 1:5 (red line), 1:10 (blue line), 1:20 (purple line). [BSA] = 0.75 μM. Figure C. Synchronous fluorescence spectra for BSA in presence of Sb(III). Synchronous fluorescence spectra for BSA in presence of Sb(III) at room temperature on dilution 1:1 (black line), 1:5 (red line), 1:10 (blue line), 1:20 (purple line). Molar ratio [BSA]:[Sb(III)] 1:10. [BSA] = 0.75 μM. Figure D. Synchronous fluorescence spectra for BSA in presence of Sb(V). Synchronous fluorescence spectra for BSA in presence of Sb(V) at room temperature on dilution 1:1 (black line), 1:5 (red line), 1:10 (blue line), 1:20 (purple line). Molar ratio [BSA]:[Sb(V)] 1:10. [BSA] = 0.75 μM. Figure E. SEC-ICP-QQQ chromatograms of BSA incubated in presence of Sb(III). SEC-ICP-QQQ chromatograms of BSA incubated by 72 hours at 37°C in presence of Sb(III) corresponding to 32S (line in black) and 121Sb (line in grey) signals. Molar ratio [BSA]:[Sb(III)] 1:10. [BSA] = 0.75 μM. Figure F. ICP-QQQ fractograms of BSA. ICP-QQQ fractograms of BSA at room temperature (black) and 58°C (grey) corresponding to the 32SO* signal. [BSA] = 0.75μM. (ZIP) [file pone.0170869.s002.zip › FigA.tif]

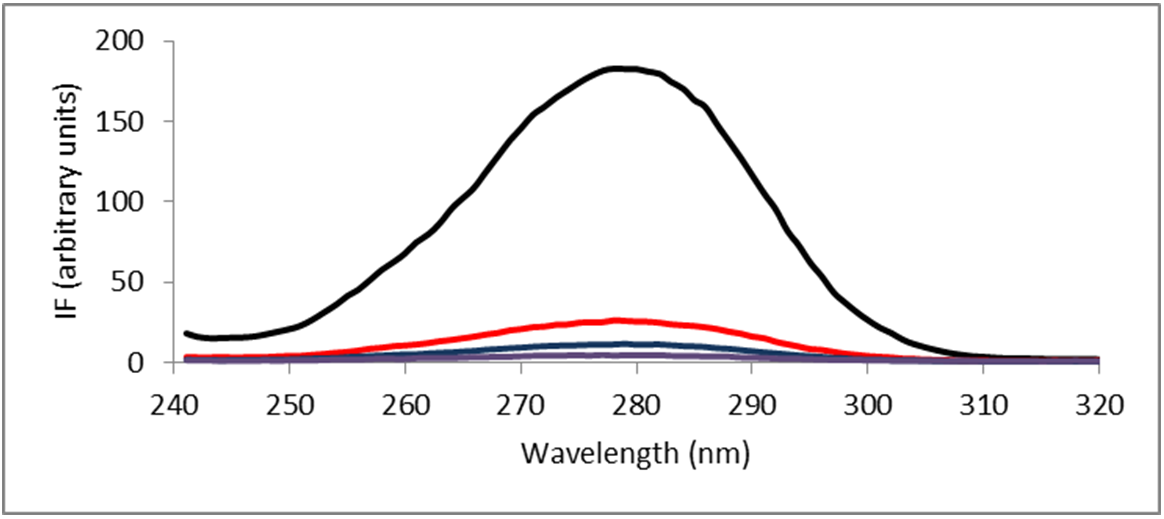

Supplement: S2 File — Figure A. BSA excitation emission matrix. [BSA] = 0.75mM. BSA excitation emission matrix in PBS at pH 7.4. [BSA] = 0.75mM. Figure B. Synchronous fluorescence spectra for BSA. [BSA] = 0.75 μM. Synchronous fluorescence spectra for BSA at room temperature on dilution 1:1 (black line), 1:5 (red line), 1:10 (blue line), 1:20 (purple line). [BSA] = 0.75 μM. Figure C. Synchronous fluorescence spectra for BSA in presence of Sb(III). Synchronous fluorescence spectra for BSA in presence of Sb(III) at room temperature on dilution 1:1 (black line), 1:5 (red line), 1:10 (blue line), 1:20 (purple line). Molar ratio [BSA]:[Sb(III)] 1:10. [BSA] = 0.75 μM. Figure D. Synchronous fluorescence spectra for BSA in presence of Sb(V). Synchronous fluorescence spectra for BSA in presence of Sb(V) at room temperature on dilution 1:1 (black line), 1:5 (red line), 1:10 (blue line), 1:20 (purple line). Molar ratio [BSA]:[Sb(V)] 1:10. [BSA] = 0.75 μM. Figure E. SEC-ICP-QQQ chromatograms of BSA incubated in presence of Sb(III). SEC-ICP-QQQ chromatograms of BSA incubated by 72 hours at 37°C in presence of Sb(III) corresponding to 32S (line in black) and 121Sb (line in grey) signals. Molar ratio [BSA]:[Sb(III)] 1:10. [BSA] = 0.75 μM. Figure F. ICP-QQQ fractograms of BSA. ICP-QQQ fractograms of BSA at room temperature (black) and 58°C (grey) corresponding to the 32SO* signal. [BSA] = 0.75μM. (ZIP) [file pone.0170869.s002.zip › FigB.tif]

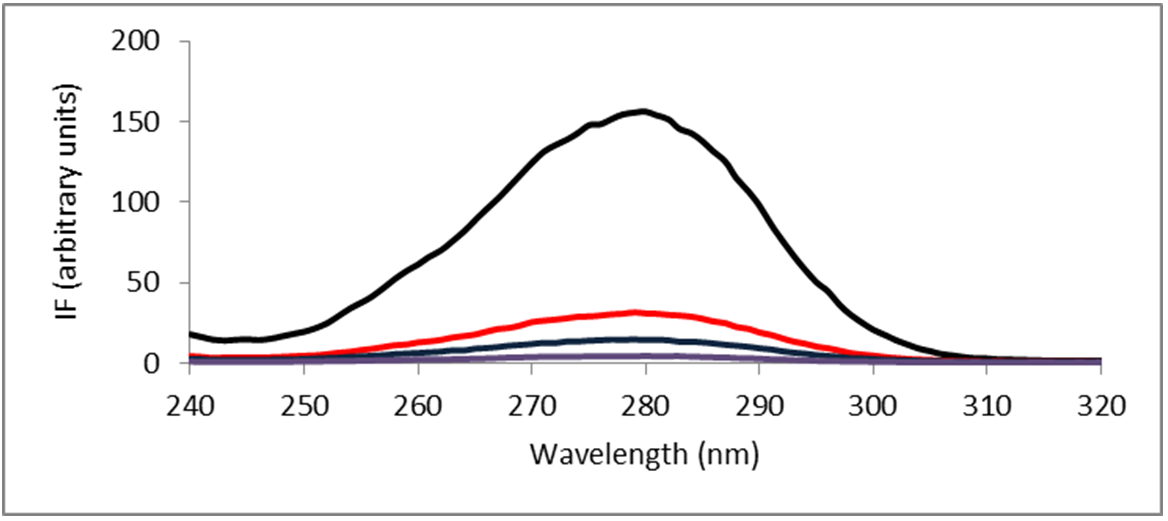

Supplement: S2 File — Figure A. BSA excitation emission matrix. [BSA] = 0.75mM. BSA excitation emission matrix in PBS at pH 7.4. [BSA] = 0.75mM. Figure B. Synchronous fluorescence spectra for BSA. [BSA] = 0.75 μM. Synchronous fluorescence spectra for BSA at room temperature on dilution 1:1 (black line), 1:5 (red line), 1:10 (blue line), 1:20 (purple line). [BSA] = 0.75 μM. Figure C. Synchronous fluorescence spectra for BSA in presence of Sb(III). Synchronous fluorescence spectra for BSA in presence of Sb(III) at room temperature on dilution 1:1 (black line), 1:5 (red line), 1:10 (blue line), 1:20 (purple line). Molar ratio [BSA]:[Sb(III)] 1:10. [BSA] = 0.75 μM. Figure D. Synchronous fluorescence spectra for BSA in presence of Sb(V). Synchronous fluorescence spectra for BSA in presence of Sb(V) at room temperature on dilution 1:1 (black line), 1:5 (red line), 1:10 (blue line), 1:20 (purple line). Molar ratio [BSA]:[Sb(V)] 1:10. [BSA] = 0.75 μM. Figure E. SEC-ICP-QQQ chromatograms of BSA incubated in presence of Sb(III). SEC-ICP-QQQ chromatograms of BSA incubated by 72 hours at 37°C in presence of Sb(III) corresponding to 32S (line in black) and 121Sb (line in grey) signals. Molar ratio [BSA]:[Sb(III)] 1:10. [BSA] = 0.75 μM. Figure F. ICP-QQQ fractograms of BSA. ICP-QQQ fractograms of BSA at room temperature (black) and 58°C (grey) corresponding to the 32SO* signal. [BSA] = 0.75μM. (ZIP) [file pone.0170869.s002.zip › FigC.tif]

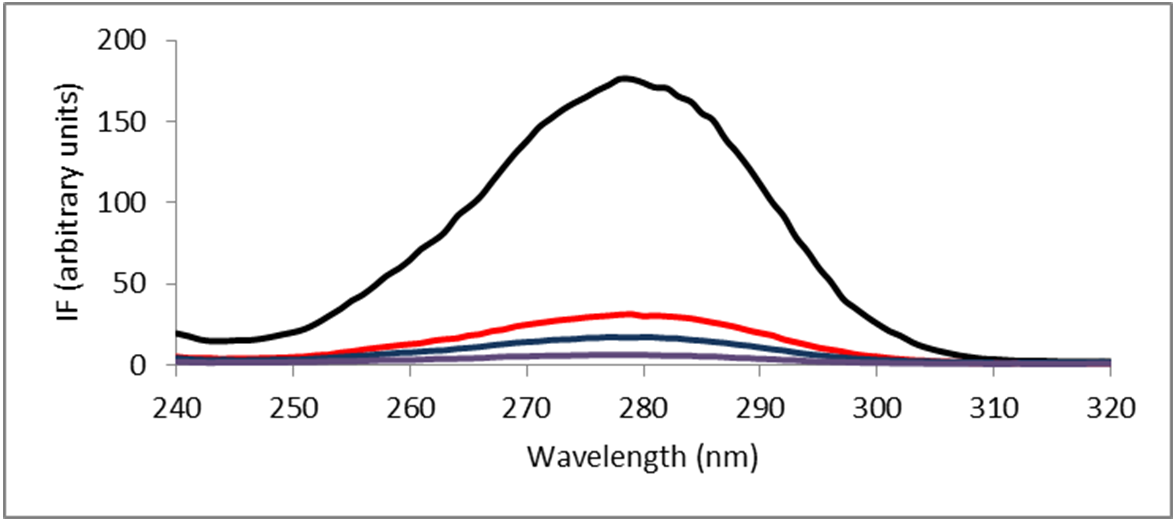

Supplement: S2 File — Figure A. BSA excitation emission matrix. [BSA] = 0.75mM. BSA excitation emission matrix in PBS at pH 7.4. [BSA] = 0.75mM. Figure B. Synchronous fluorescence spectra for BSA. [BSA] = 0.75 μM. Synchronous fluorescence spectra for BSA at room temperature on dilution 1:1 (black line), 1:5 (red line), 1:10 (blue line), 1:20 (purple line). [BSA] = 0.75 μM. Figure C. Synchronous fluorescence spectra for BSA in presence of Sb(III). Synchronous fluorescence spectra for BSA in presence of Sb(III) at room temperature on dilution 1:1 (black line), 1:5 (red line), 1:10 (blue line), 1:20 (purple line). Molar ratio [BSA]:[Sb(III)] 1:10. [BSA] = 0.75 μM. Figure D. Synchronous fluorescence spectra for BSA in presence of Sb(V). Synchronous fluorescence spectra for BSA in presence of Sb(V) at room temperature on dilution 1:1 (black line), 1:5 (red line), 1:10 (blue line), 1:20 (purple line). Molar ratio [BSA]:[Sb(V)] 1:10. [BSA] = 0.75 μM. Figure E. SEC-ICP-QQQ chromatograms of BSA incubated in presence of Sb(III). SEC-ICP-QQQ chromatograms of BSA incubated by 72 hours at 37°C in presence of Sb(III) corresponding to 32S (line in black) and 121Sb (line in grey) signals. Molar ratio [BSA]:[Sb(III)] 1:10. [BSA] = 0.75 μM. Figure F. ICP-QQQ fractograms of BSA. ICP-QQQ fractograms of BSA at room temperature (black) and 58°C (grey) corresponding to the 32SO* signal. [BSA] = 0.75μM. (ZIP) [file pone.0170869.s002.zip › FigD.tif]

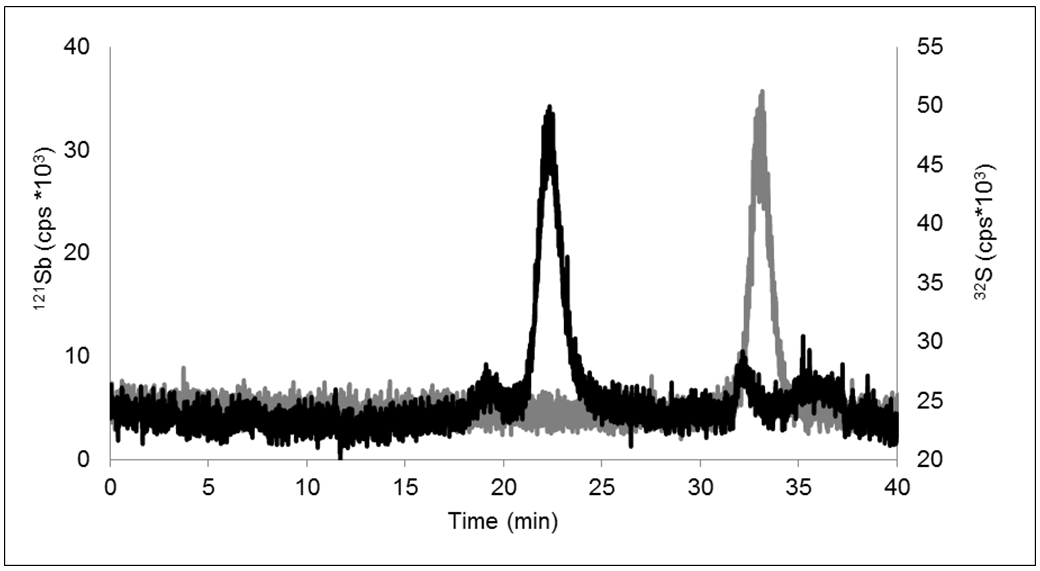

Supplement: S2 File — Figure A. BSA excitation emission matrix. [BSA] = 0.75mM. BSA excitation emission matrix in PBS at pH 7.4. [BSA] = 0.75mM. Figure B. Synchronous fluorescence spectra for BSA. [BSA] = 0.75 μM. Synchronous fluorescence spectra for BSA at room temperature on dilution 1:1 (black line), 1:5 (red line), 1:10 (blue line), 1:20 (purple line). [BSA] = 0.75 μM. Figure C. Synchronous fluorescence spectra for BSA in presence of Sb(III). Synchronous fluorescence spectra for BSA in presence of Sb(III) at room temperature on dilution 1:1 (black line), 1:5 (red line), 1:10 (blue line), 1:20 (purple line). Molar ratio [BSA]:[Sb(III)] 1:10. [BSA] = 0.75 μM. Figure D. Synchronous fluorescence spectra for BSA in presence of Sb(V). Synchronous fluorescence spectra for BSA in presence of Sb(V) at room temperature on dilution 1:1 (black line), 1:5 (red line), 1:10 (blue line), 1:20 (purple line). Molar ratio [BSA]:[Sb(V)] 1:10. [BSA] = 0.75 μM. Figure E. SEC-ICP-QQQ chromatograms of BSA incubated in presence of Sb(III). SEC-ICP-QQQ chromatograms of BSA incubated by 72 hours at 37°C in presence of Sb(III) corresponding to 32S (line in black) and 121Sb (line in grey) signals. Molar ratio [BSA]:[Sb(III)] 1:10. [BSA] = 0.75 μM. Figure F. ICP-QQQ fractograms of BSA. ICP-QQQ fractograms of BSA at room temperature (black) and 58°C (grey) corresponding to the 32SO* signal. [BSA] = 0.75μM. (ZIP) [file pone.0170869.s002.zip › FigE.tif]

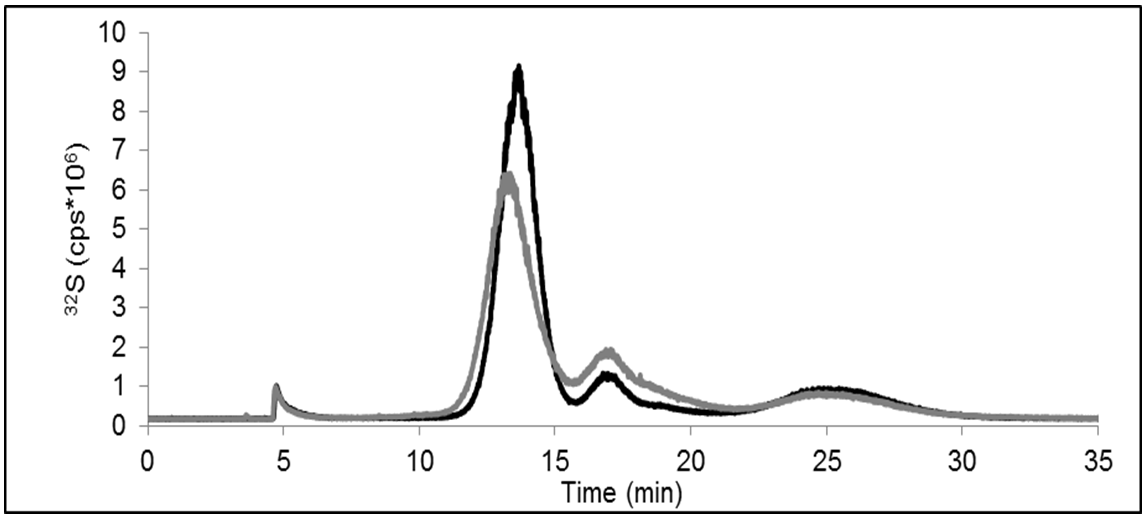

Supplement: S2 File — Figure A. BSA excitation emission matrix. [BSA] = 0.75mM. BSA excitation emission matrix in PBS at pH 7.4. [BSA] = 0.75mM. Figure B. Synchronous fluorescence spectra for BSA. [BSA] = 0.75 μM. Synchronous fluorescence spectra for BSA at room temperature on dilution 1:1 (black line), 1:5 (red line), 1:10 (blue line), 1:20 (purple line). [BSA] = 0.75 μM. Figure C. Synchronous fluorescence spectra for BSA in presence of Sb(III). Synchronous fluorescence spectra for BSA in presence of Sb(III) at room temperature on dilution 1:1 (black line), 1:5 (red line), 1:10 (blue line), 1:20 (purple line). Molar ratio [BSA]:[Sb(III)] 1:10. [BSA] = 0.75 μM. Figure D. Synchronous fluorescence spectra for BSA in presence of Sb(V). Synchronous fluorescence spectra for BSA in presence of Sb(V) at room temperature on dilution 1:1 (black line), 1:5 (red line), 1:10 (blue line), 1:20 (purple line). Molar ratio [BSA]:[Sb(V)] 1:10. [BSA] = 0.75 μM. Figure E. SEC-ICP-QQQ chromatograms of BSA incubated in presence of Sb(III). SEC-ICP-QQQ chromatograms of BSA incubated by 72 hours at 37°C in presence of Sb(III) corresponding to 32S (line in black) and 121Sb (line in grey) signals. Molar ratio [BSA]:[Sb(III)] 1:10. [BSA] = 0.75 μM. Figure F. ICP-QQQ fractograms of BSA. ICP-QQQ fractograms of BSA at room temperature (black) and 58°C (grey) corresponding to the 32SO* signal. [BSA] = 0.75μM. (ZIP) [file pone.0170869.s002.zip › FigF.tif]
